# Clinical Trial of Salmon Nasal Cartilage‐Derived Proteoglycans on Human Facial Antiaging: A Randomized, Double‐Blind, Placebo‐Controlled Study

**DOI:** 10.1111/jocd.70218

**Published:** 2025-07-04

**Authors:** Xue‐dong Bai, Yu‐chen Liu, Sai‐ya Ge, Wei‐cheng Fei

**Affiliations:** ^1^ R&D Center of Shanghai Huiwen Biotech Co., Ltd Shanghai People's Republic of China

**Keywords:** cartilage extract, hydration, pigmentation, proteoglycan, skin elasticity, wrinkles

## Abstract

**Background:**

Proteoglycans (PGs) derived from salmon nasal cartilage are believed to have antiaging effects on the skin. However, comprehensive evaluations of their impact on various skin parameters in Chinese populations remain limited.

**Aims:**

This study aims to evaluate the efficacy of oral PG supplementation in enhancing skin elasticity, hydration, and reducing roughness, wrinkles, and pigmentation in healthy adult volunteers.

**Methods:**

A 56‐day randomized, double‐blind, placebo‐controlled trial was conducted involving 66 subjects aged 30–60. Subjects received a daily dose of 20 mg PG, and skin parameters were measured at baseline, 28 days, and 56 days. The study assessed skin elasticity, hydration, roughness, wrinkles, melanin content, and brown spots while monitoring for any adverse effects.

**Results:**

Subjects receiving PG supplementation showed significant improvements in skin elasticity and hydration at both 28 days and 56 days (*p* < 0.001), with reductions in skin roughness and wrinkles (*p* < 0.001), and a significant decrease in melanin content and brown spots (*p* < 0.001). Compared to the placebo group, the PG group exhibited significant improvements in most skin parameters by 56 days, except in the wrinkle area percentage at the crow's feet, where no significant difference was observed. PG was well tolerated, with no adverse effects reported.

**Conclusions:**

Our findings suggest that daily oral intake of 20 mg PG effectively improves skin health by enhancing elasticity, hydration, and reducing signs of aging such as wrinkles and pigmentation.

## 1 Introduction

Skin aging can be classified into two types based on exogenous and endogenous mechanisms: exogenous aging caused by factors such as harmful chemicals, air pollution, and primarily photoaging, and internal aging, which results from the natural aging process [1]. Endogenous aging is driven by various environmental factors, such as air pollution, lifestyle stress, irregular sleep patterns, and UV radiation exposure [2], with UV radiation playing a significant role by inducing oxidative stress, DNA damage, and inflammation [3, 4]. Endogenous aging, on the other hand, is influenced by genetic factors that regulate cellular senescence [5], telomere shortening [6], and decreased cellular repair mechanisms [7]. Both types of aging commonly damage the extracellular matrix in the dermis, particularly collagen [8], leading to visible skin changes such as pigmentation, thinning of the skin barrier, accelerated formation of wrinkles, and loss of skin elasticity [9]. As a result, the search for functional foods that can delay skin aging has become an increasingly popular topic among consumers [10].

Proteoglycans derived from salmon nasal cartilage, with a long history of use in Japan, are now commercially utilized for promoting joint health and skin benefits [11, 12]. Current extraction methods include those published by Shigemitsu Kudo et al., involving ethanol extraction of dried salmon nasal cartilage [13], and by Majima et al., using acetic acid extraction of frozen salmon nasal cartilage slices [14]. Yota Tatara et al. reported a method involving micronization of salmon nasal cartilage in ethanol using a rotor‐stator homogenizer, followed by extraction with magnesium chloride and purification through anion exchange chromatography [15]. However, this method does not describe its applicability for industrial‐scale production. In our published patent, we describe a novel method suitable for industrial production [16]. Briefly, this method uses enzymatic extraction with water under different temperature gradients, then is centrifuged and separated through ultrafiltration membranes. Finally, the separated liquid undergoes freeze‐drying to obtain the proteoglycan product.

Proteoglycans are widely distributed in human skin, cartilage, blood vessels, and other connective tissues [17]. In the skin, proteoglycans, such as decorin, are critical for maintaining the structural integrity and functionality of the extracellular matrix by binding to collagen, protecting the fibrous structures, and enhancing the effects of TGF‐β [18, 19]. Although proteoglycans derived from salmon nasal cartilage differ structurally from human proteoglycans, structural studies support their potential in antiaging. Research indicates that proteoglycans derived from salmon nasal cartilage are composed of type II collagen and glycosaminoglycans, such as chondroitin sulfate. These glycosaminoglycans are covalently linked to specific serine residues within the core protein via a tetrasaccharide linker (glucuronic acid‐1,3‐galactose‐1,3‐galactose‐1,4‐xylose‐1), and the G3 domain exhibits significant sequence similarity with human EGF1 [20].

Current clinical studies on the oral cosmetic benefits of proteoglycans include research by Tatsuji Takahashi et al., which investigated the effects of oral PG supplementation on reducing transepidermal water loss and improving skin hydration in subjects [21]. Additionally, human evaluations were conducted on skin elasticity, wrinkles, facial pores, blotches, moisture, and smoothness [22]. However, these studies were conducted exclusively on Japanese subjects, with a lack of research on Chinese populations. Moreover, previous studies had limitations, such as the specific types of data collection equipment used and the relatively small sample sizes. Due to variations in product manufacturing processes, as mentioned earlier, clinical research specific to the proteoglycan product described in this study is necessary to explore its efficacy. Addressing these gaps, we conducted a randomized, double‐blind, placebo‐controlled clinical trial involving Chinese subjects, using industry‐standard instruments such as VISIA‐CR, CK‐MPA10, and Antera‐3D to collect facial data. This study aimed to further validate the antiaging effects of orally administered proteoglycans derived from salmon nasal cartilage on human skin by examining the external phenotypes of facial skin aging.

## 2 Materials and Methods

### 2.1 Product

Salmon Nasal Cartilage‐Derived Proteoglycans was provided by Shanghai Huiwen Biotech Corp. Ltd. (Shanghai, China), Lot No. 231115. Other excipients include maltodextrin (Aladdin), green tea flavored powder essence F‐7248 (Shanghai Fangjing Flavors & Fragrances Co. Ltd.), and sucralose (JK Sucralose Inc.). The salmon nasal cartilage powder was analyzed using methods published by the National Institutes for Food and Drug Control of China and the Chinese corporate standard Q/310115 HWSW 0001‐2024. The results were as follows: moisture loss on drying 5.68%, ash content 10.58%, chondroitin sulfate (as dry base) 40.65%, and type II collagen (as dry base) 40.79%. The content of chondroitin sulfate and proteoglycan was determined using the barium sulfate gravimetric method as described in the Chinese corporate standard Q/310115 HWSW 0001‐2024. The content calculations are shown in Equations (1) and (2):

(1) $$\begin{array}{cc} \text{Chondroitin sulfate}\;\left(\%\right)= & \left(\text{Barium sulfate}\;\left(g\right)\times 503.3\right)/\\ & \left(\text{Testing samples}\;\left(g\right)\times 233.4\right)\times 100\% \end{array}$$

(2) $$\text{Type}\;\mathrm{II}\;\text{collagen}\;\left(\%\right)=\text{Chondroitin sulfate}\;\left(\%\right)\times 1.075$$

**FIGURE 1:**
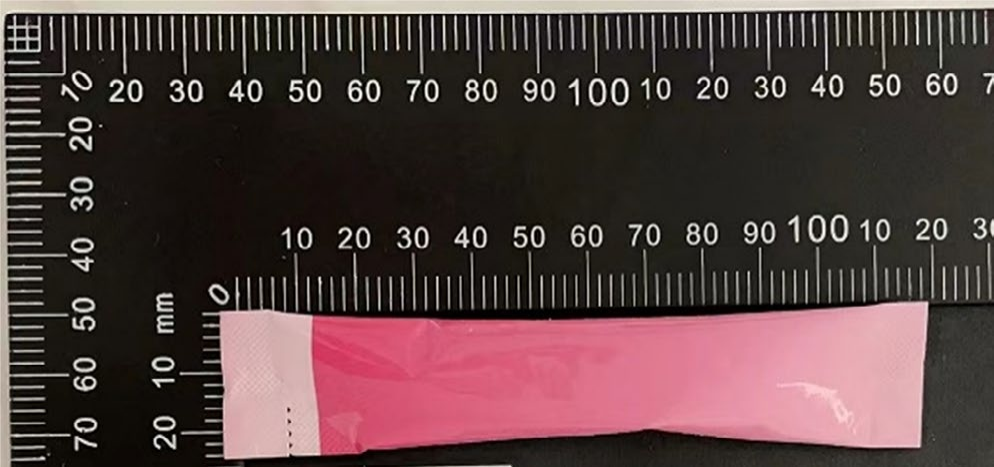
Sample photo of PG solid beverage.

The above materials were mixed according to the ratios in Table 1 and then packaged in sealed strip packs, each containing 5 g. The sample photo of PG solid beverage is shown in Figure 1. The products were divided into two groups: one was the placebo group, which contained no PG, and the other was the PG group, which contained 0.02 g of PG. Subjects received identically packaged products according to their assigned groups and were instructed to take one portion daily.

**TABLE 1:** PG solid beverage ingredient list.

Ingredient	Daily intake (g)	
	Placebo	PG
PG	/	0.02
Maltodextrin	4.763	4.563
Green tea flavor powder essence	0.4	0.4
Sucralose trichloride	0.017	0.017
Total	5	5

### 2.2 Subjects

The study was a randomized, double‐blind, placebo‐controlled trial conducted at Shanghai Huiwen Biotech Corp. Ltd. (Shanghai, China). Subjects, including healthy males and females aged 31–57, were recruited from Shanghai. Huiwen has an independent third‐party laboratory that conducts in vitro, in vivo, chemical, microbiological, and clinical tests on products, including cosmetics, food, and nutraceuticals. Subjects were required to have no history of participation in other clinical studies within the last 3 months, to be subjectively cooperative with the study procedures, and to maintain their usual lifestyle during the study. All procedures were conducted in accordance with the World Medical Association's (WMA) Declaration of Helsinki and its subsequent amendments (Ethical Principles for Medical Research Involving Human Subjects, adopted by the 18th WMA General Assembly, Helsinki, Finland, June 1964, with subsequent amendments). The study protocol was reviewed and approved by Huiwen's institutional review board under Protocol No. Huiwen BIO_202312A066. On the first day of the study, all subjects were invited to visit Huiwen for screening and enrollment. All follow‐up clinical assessments were conducted at Huiwen. Subjects were fully informed about the study procedures, provided with the informed consent form, and voluntarily signed it after reading and understanding its content. Each enrolled subject was assigned a unique identification number through a computer‐generated randomization process, ensuring balanced group allocation. The study was conducted in a double‐blind manner, meaning that both the subjects and the assessors were unaware of the group assignments.

### 2.3 Screening Criteria for Subjects

Subjects will be excluded from participation if they meet any of the following criteria: use of systemic antihistamines within the past week, use of systemic immunosuppressants, topical hydroxy acids, skin‐whitening agents, or antiaging treatments within the past month, facial use of anti‐inflammatory medications within the past 2 months, unresolved inflammatory skin conditions, insulin‐dependent diabetes, history of bilateral axillary lymph node dissection, being under treatment for asthma or other chronic respiratory diseases, having undergone cancer chemotherapy within the past 6 months, currently receiving dermatological treatment, or having extensive skin conditions in the test area such as large birthmarks, scratches, vitiligo, pigmented nevi, or keloid scars that could affect the trial results. Additionally, individuals deemed unsuitable for this trial by the principal investigator will also be excluded.

### 2.4 Study Procedure

This study was conducted from December 19, 2023, to February 23, 2024. After signing informed consent forms, subjects cleaned their faces and then rested for 30 min in a controlled environment with a temperature of 21°C ± 1°C and humidity of 50% ± 10% RH. The controlled environment was essential to minimize external variables that could affect skin measurements. Subjects were randomly assigned to two groups in a double‐blind manner, ensuring that neither the subjects nor the testers knew the group allocations. The testers used a series of calibrated instruments to measure the baseline skin values of the subjects' cheeks, with all follow‐up measurements taken from the same locations. Proper calibration of all instruments ensured the accuracy and consistency of the skin measurements across all subjects.

The moisture content of the stratum corneum was measured using the Corneometer CM825 (Courage + Khazaka, Germany), a noninvasive capacitance probe. The instrument operates on the capacitance method, measuring capacitance based on a frequency shift in the applied oscillating system [23]. Skin elasticity (R2) was assessed using the Cutometer MPA 580 (Courage + Khazaka, Germany), which employs a time/strain mode for 18 s followed by a two‐second relaxation period, using a 2 mm probe that applies a constant suction pressure of 350 mbar [24]. Melanin levels were determined using the Mexameter MX18(Courage + Khazaka, Germany), based on the narrowband reflectance spectrophotometer principle. This device emits wavelengths of 660 nm and 880 nm and measures melanin levels by assessing the intensity of reflected light [25]. The above three types of detection probes are assembled on the CK‐MPA10 system(Courage + Khazaka, Germany).

The areas and percentages of crow's feet, under‐eye wrinkles, and skin brown spots were measured using the VISIA‐CR system (Canfield Scientific). Subjects were instructed to close their eyes and maintain a consistent photographing angle while facial images were captured in Parallel‐Polarized mode and Cross‐Polarized mode with RBX Technology [26]. These images were then analyzed quantitatively using Image Plus Pro software (Media Cybernetics).

The skin roughness of a 5.6 × 5.6 cm^2^ area on the participant's cheek was measured using the Antera 3D system. Antera 3D is a handheld camera equipped with multi‐LED technology and accompanying software that ensures consistent area measurement for accurate longitudinal comparison. The system measures the roughness score (Ra) within a circular area of 2.85 cm in diameter. The Ra score is determined by analyzing the peaks and valleys on the surface using a set of 24 images captured from different angles with the Antera 3D camera [27].

Each subject was provided with 56 packets of the solid beverage and instructed to consume one packet daily, dissolved in warm water half an hour after a meal. Subjects were required to maintain their usual diet and exercise habits throughout the study, avoiding irregular lifestyle changes such as disrupted sleep patterns, altering cosmetic or skincare products, beauty treatments, and excessive UV exposure. Subjects were also given questionnaires to be completed at the 28‐day and 56‐day marks, assessing their satisfaction with six different aspects on a scale from 0 (least satisfied) to 5 (most satisfied). Subjects returned for follow‐up visits on Days 28 and 56, during which the same skin measurements were taken, and the completed questionnaires were collected to calculate the average score for each question.

### 2.5 Adverse Events

In this study, adverse reactions were monitored by instructing subjects to report any discomfort or symptoms throughout the trial. Regular assessments were conducted at baseline, Day 28, and Day 56 to check for skin irritation, gastrointestinal issues, or other health concerns. Throughout the study, subjects were also asked to maintain a diary of any adverse effects.

### 2.6 Statistical Analysis

Statistical analyses were conducted using IBM Statistical Package for the Social Sciences (SPSS 21.0) software. Skin metric data were expressed as mean ± SEM, and normality was assessed. If the data met the normal distribution requirements, paired *t*‐tests were used for pre‐ and posttreatment comparisons; otherwise, the Wilcoxon signed‐rank test was used. For ordinal data, pre‐ and posttreatment comparisons were also made using the Wilcoxon signed‐rank test. Comparisons between the test product and control groups were conducted using two‐way ANOVA. All statistical analyses were two‐tailed, with a significance level of *α* = 0.05.

## 3 Results and Discussion

### 3.1 Project Completion Status

**FIGURE 2:**
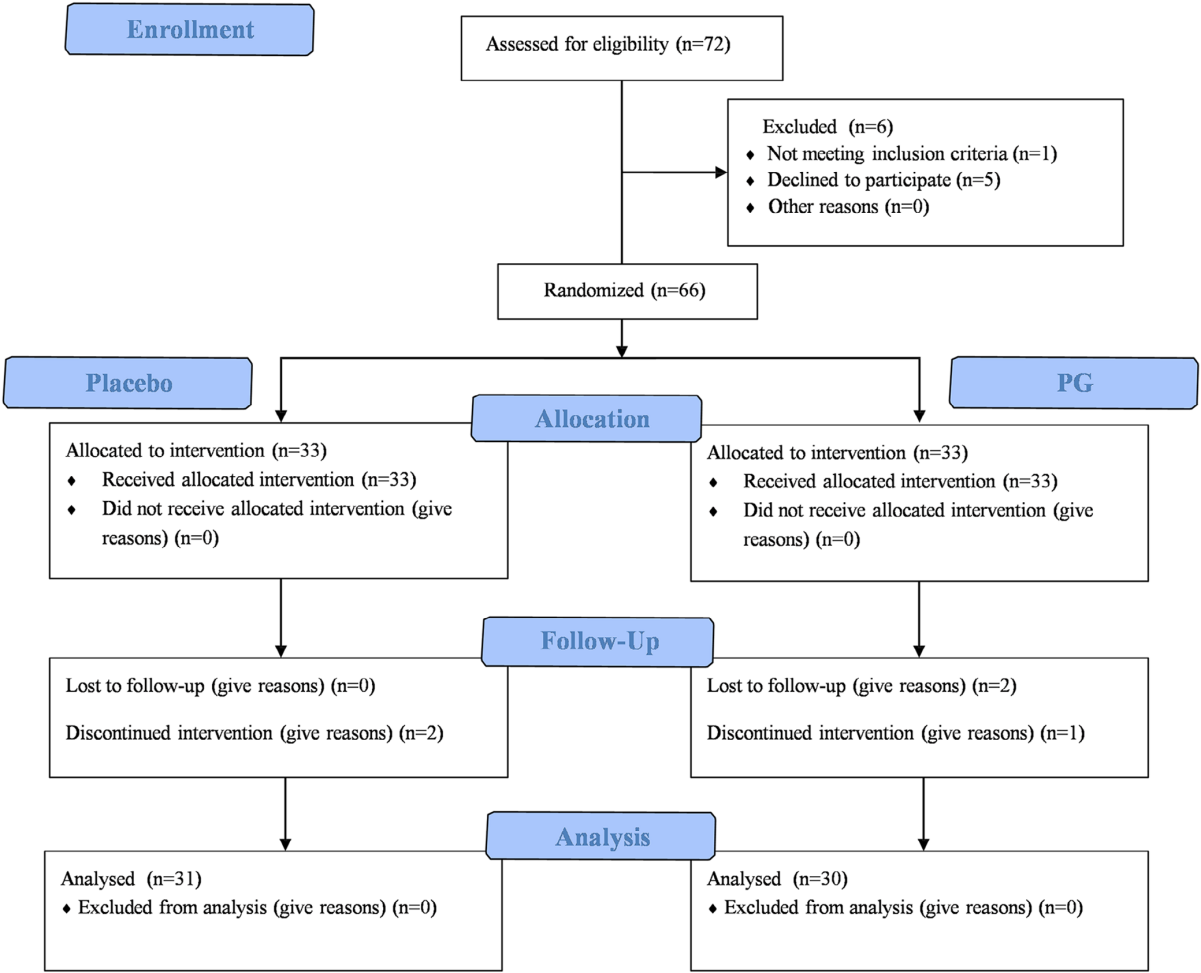
CONSORT flow diagram of this study.

A total of 72 volunteers meeting the screening criteria were enrolled in this long‐term study. One individual did not attend the informed consent meeting, and five subjects voluntarily withdrew after reviewing the study details, leaving 66 subjects who completed the study. These subjects were randomly assigned in a double‐blind manner to either the placebo group or the PG group, with 33 subjects in each group. In the placebo group, two subjects did not adhere to the product usage guidelines, resulting in 31 usable data sets, with an average age of 40.39 ± 6.01 years. In the PG group, one subject did not adhere to the product usage guidelines, and two subjects voluntarily withdrew, resulting in 30 usable data sets, with an average age of 45.73 ± 5.58 years. Recruitment details and demographic information are provided in Figure 2 and Table 2. The baseline values of various skin parameters for both groups are provided in Table 3. No adverse reactions such as skin redness, itching, dryness, flaking, sensitivity, or gastrointestinal distress were observed in any subjects.

**TABLE 2:** Subject participant allocation and demographic characteristics.

Parameter		Placebo	PG
Allocated numbers		33	33
Drop numbers		2	3
Analysis numbers		31	30
Age (Years)		40.39 ± 6.01^a^	45.73 ± 5.58^a^
Sex	Female	28	29
	Male	3	1

^a^ Statistical data are presented as Mean ± SEM.

**TABLE 3:** Statistics of baseline values for subjects' skin indicators.

Parameter	Placebo^a^	PG^a^
Skin hydration	40.35 ± 2.28	42.80 ± 2.19
Skin elasticity R2	0.53 ± 0.01	0.54 ± 0.01
Skin roughness Ra	6.51 ± 0.29	6.79 ± 0.32
Crow's feet area	2.29 ± 0.18	2.34 ± 0.22
The proportion of crow's feet area	6.22 ± 0.74	6.40 ± 0.64
Lower eyelid wrinkles area	3.57 ± 0.25	3.49 ± 0.28
The proportion of Lower eyelid wrinkles area	13.90 ± 0.93	13.63 ± 1.19
Skin heme	141.83 ± 7.69	151.11 ± 6.67
Skin brown spots area	22.28 ± 1.07	23.92 ± 0.94
The proportion of Skin brown spots area	43.72 ± 1.99	44.88 ± 1.85

^a^ Statistical data are presented as Mean ± SEM.

### 3.2 Effects of Salmon Nasal Cartilage Proteoglycans on Skin Moisture, Elasticity, and Roughness

**FIGURE 3:**
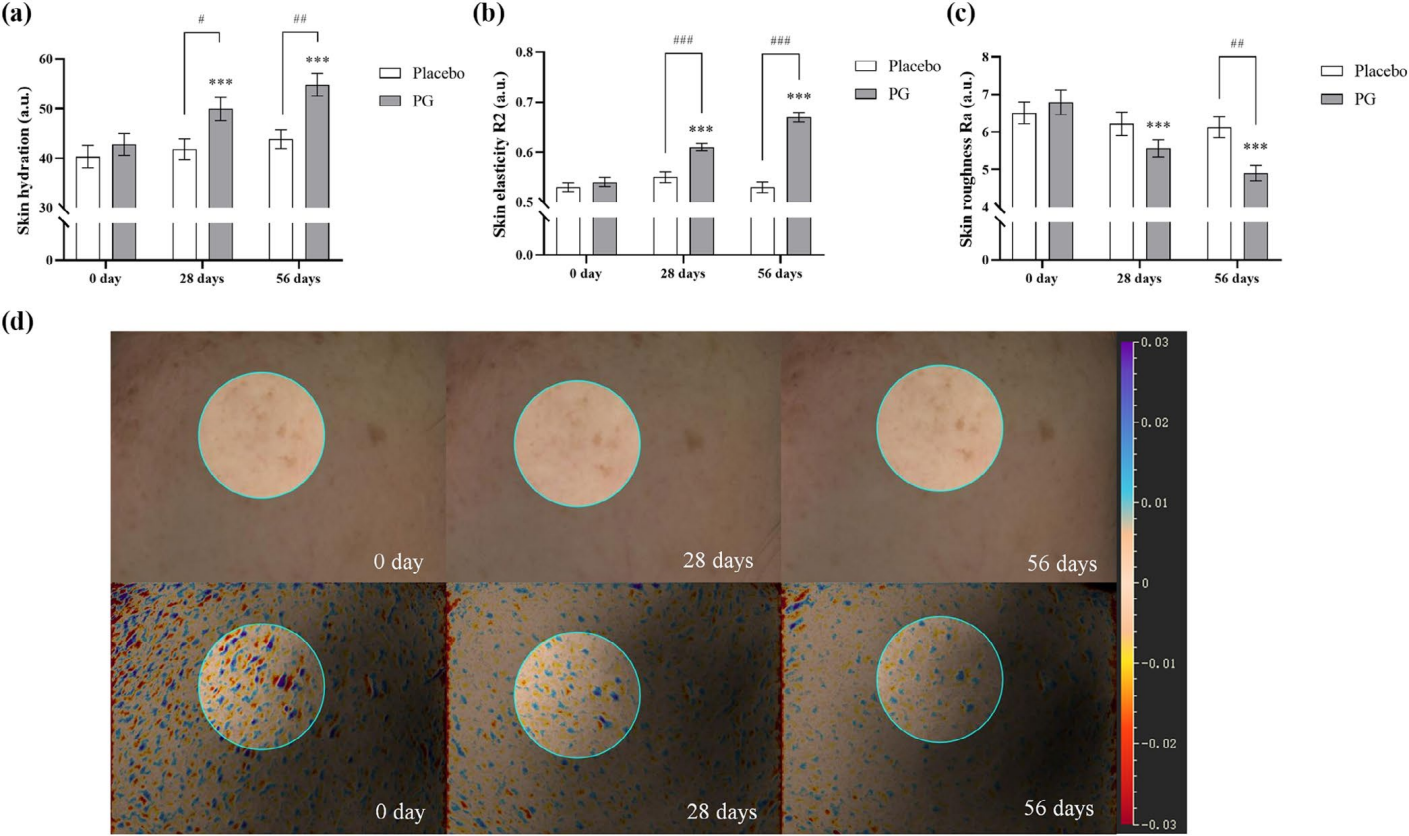
Effects of PG and placebo on skin moisture, elasticity, and roughness after 28 and 56 Days. Baseline skin parameters were measured, and subjects consumed PG or placebo for 56 days, with follow‐up assessments on Days 28 and 56. (a) Skin moisture content was measured on Days 0, 28, and 56 using the Corneometer CM825. (b) Skin elasticity (R2) was assessed on Days 0, 28, and 56 using the Cutometer dual MPA580. (c) Skin roughness (Ra) was measured on Days 0, 28, and 56 using Antera‐3D, which analyzed 2D texture images. (d) An example of images and 2D color and texture analysis from Antera‐3D, with the analysis area highlighted by a circular region and skin texture roughness indicated by the standard color reference on the far right. Data are expressed as mean ± SEM. Statistical significance: ***p < 0.001 (within‐group changes in the PG group compared to the baseline value, paired *t*‐test); #*p* < 0.05, ##*p* < 0.01, ###*p* < 0.001 (between‐group changes between the PG and placebo groups at the same time point, two‐way ANOVA).

The CK‐MPA10 skin analysis system was used to assess skin moisture content and skin elasticity (R2) in the subjects' cheek areas, whereas the Antera‐3D system was utilized for imaging and analyzing skin roughness (Ra). Results showed a statistically significant increase in skin moisture content (Figure 3a), skin elasticity (R2) (Figure 3b), and a reduction in skin roughness (Ra) (Figure 3c) in the PG group compared to baseline values. A representative image of skin roughness measurement in subjects is shown in Figure 3d. The statistical results of the data in this study are presented in Table 4. Compared to the placebo group, the PG group exhibited statistically significant improvements in skin moisture content and skin elasticity (R2) by Day 28, showing improvement rates of 13.06% ([95% CI of diff.: −15.560 to −0.700], *p* < 0.05) and 9.19% ([95% CI of diff.: −0.092 to −0.0278], *p* < 0.001), respectively. By Day 56, further significant improvements were observed in skin moisture content, skin elasticity (R2), and skin roughness (Ra), with improvement rates of 19.35% ([95% CI of diff.: −18.380 to −3.520], *p* < 0.01), 24.08% ([95% CI of diff.: −0.172 to −0.108], *p* < 0.001), and 21.85% ([95% CI of diff.: 0.273 to 2.167], *p* < 0.01) compared to the placebo group.

**TABLE 4:** Comparison of skin parameters between placebo and PG groups at 28 and 56 Days.

Parameter	28 days				56 days			
	Placebo		PG		Placebo		PG	
	Value^a^	Rate	Value^a^	Rate	Value^a^	Rate	Value^a^	Rate
Skin hydration	41.84 ± 2.12	3.69%	49.97 ± 2.31***	16.75%	43.90 ± 1.18	8.80%	54.85 ± 2.27***	28.15%
Skin elasticity R2	0.55 ± 0.01	3.77%	0.61 ± 0.01***	12.96%	0.53 ± 0.01	−0.01%	0.67 ± 0.01***	24.07%
Skin roughness Ra	6.22 ± 0.31	−4.45%	5.57 ± 0.23***	−17.97%	6.13 ± 0.28	−5.84%	4.91 ± 0.21***	−27.69%
Crow's feet area	2.42 ± 0.21	5.68%	1.93 ± 0.19***	−17.35%	2.39 ± 0.17	4.37%	1.73 ± 0.20***	−26.13%
The proportion of crow's feet area	6.24 ± 0.57	0.32%	5.29 ± 0.52***	−17.38%	6.31 ± 0.53	1.45%	4.73 ± 0.52***	−26.17%
Lower eyelid wrinkles area	3.38 ± 0.25	−5.16%	2.88 ± 0.24***	−17.31%	3.42 ± 0.23	−4.04%	2.48 ± 0.18***	−28.97%
The proportion of lower eyelid wrinkles area	13.53 ± 0.84	−2.61%	11.31 ± 0.97***	−17.02%	12.98 ± 0.88	−6.56%	9.70 ± 0.76***	−28.89%
Skin heme	138.37 ± 6.35	−2.44%	127.98 ± 6.10***	−15.31%	136.74 ± 6.86	−3.59%	111.64 ± 5.97***	−26.12%
Skin brown spots area	22.68 ± 1.29	1.82%	20.94 ± 1.13***	−12.46%	23.62 ± 1.10	6.02%	19.10 ± 1.14***	−20.12%
The proportion of skin brown spots area	42.97 ± 1.81	−1.70%	39.41 ± 2.13***	−12.19%	43.17 ± 2.05	−1.24%	35.79 ± 2.13***	−20.26%

*Note:* Statistical difference expression: ***p < 0.001, indicating within‐group changes in the PG group compared to the baseline value (paired *t*‐test).

^a^ Statistical data are presented as Mean ± SEM.

### 3.3 Effects of Salmon Nasal Cartilage Proteoglycans on Improving Periorbital Wrinkles

**FIGURE 4:**
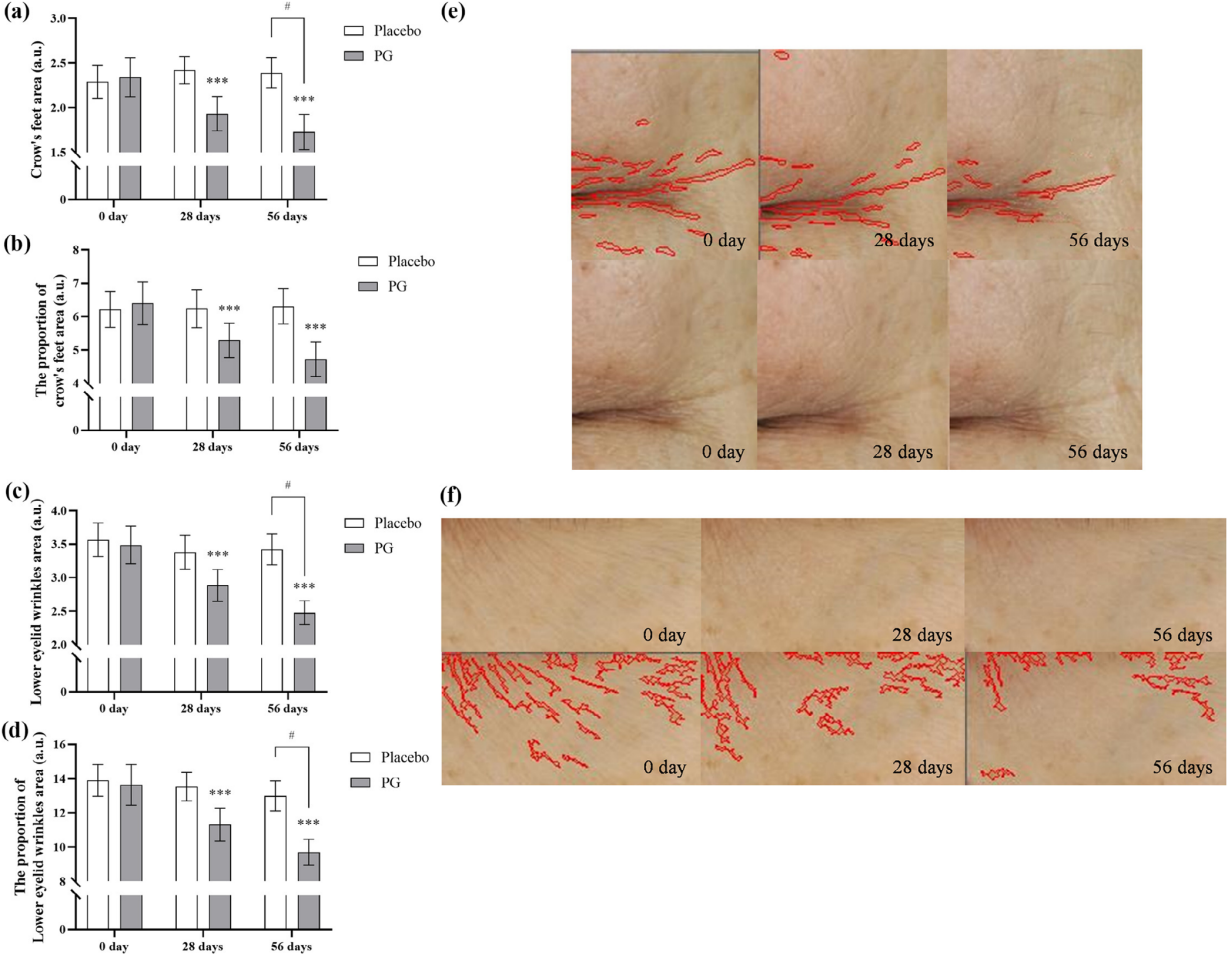
Effects of PG and placebo on periorbital wrinkles improvement after 28 and 56 days. Baseline skin parameters were measured, and subjects consumed PG or placebo for 56 days, with follow‐up assessments on Days 28 and 56. Facial images were captured using the VISIA‐CR system to measure crow's feet area (a), crow's feet area percentage (b), under‐eye wrinkle area (c), and under‐eye wrinkle area percentage (d) on Days 0, 28, and 56. Example images of crow's feet (e) and under‐eye wrinkles (f) were captured with VISIA‐CR, with wrinkles highlighted in red using Image Plus Pro software. Data are expressed as mean ± SEM. Statistical significance: ***p < 0.001 (within‐group changes in the PG group compared to the baseline value, paired *t*‐test); #*p* < 0.05, ##*p* < 0.01, ###*p* < 0.001 (between‐group changes between the PG and placebo groups at the same time, two‐way ANOVA).

Frontal facial images of the subjects were captured using the Parallel‐Polarized mode of the VISIA‐CR system. Image‐PRO Plus (Media Cybernetics) was used to select and analyze fixed areas for wrinkle area and wrinkle area percentage. Results showed significant reductions in the crow's feet area (Figure 4a) and area percentage (Figure 4b), as well as reductions in the under‐eye wrinkle area (Figure 4c) and area percentage (Figure 4d) compared to baseline values in the PG group. Representative images of crow's feet (Figure 4e) and under‐eye wrinkles (Figure 4f) from the PG group were marked with red lines, indicating visible improvements in wrinkle length, depth, and width. Compared to the placebo control, after 56 days of product use, the PG group showed statistically significant improvements in crow's feet area, under‐eye wrinkle area, and under‐eye wrinkle area percentage compared to the placebo group, with improvement rates of 21.76% ([95% CI of diff.: 0.028 to 1.295], *p* < 0.05), 33.11% ([95% CI of diff.: 0.122 to 1.765], *p* < 0.05), and 35.45% ([95% CI of diff.: 0.097 to 6.478], *p* < 0.05), respectively.

### 3.4 Effects of Salmon Nasal Cartilage Proteoglycans on Reducing Skin Pigmentation

**FIGURE 5:**
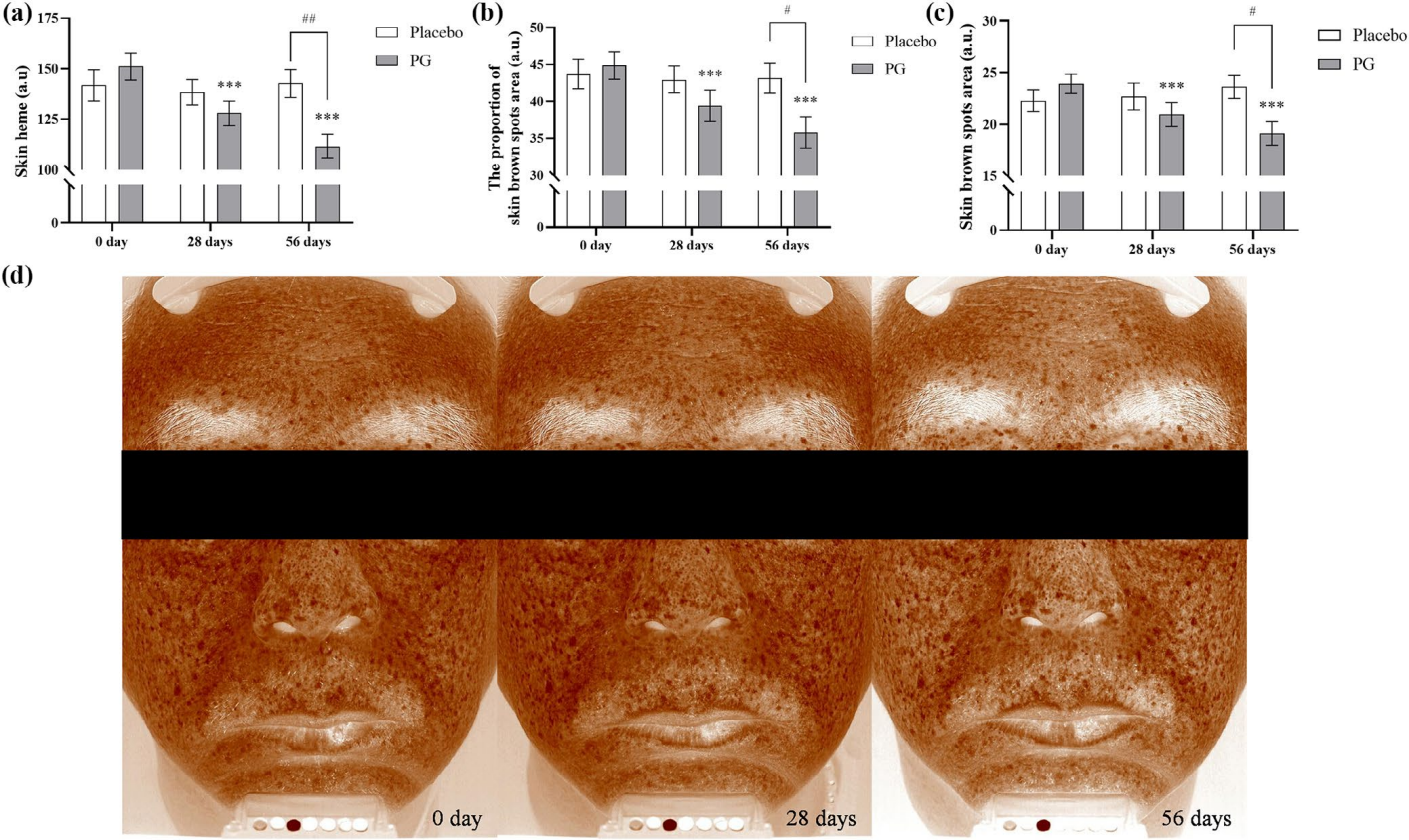
Effects of PG and placebo on reducing skin pigmentation after 28 and 56 Days. Baseline skin parameters were measured, and subjects consumed PG or placebo for 56 days, with follow‐up assessments on Days 28 and 56. (a) Melanin levels was measured on Days 0, 28, and 56 using the Mexameter MX18. (b) Skin brown spots area was measured on Days 0, 28, and 56 using the VISIA‐CR system. (c) Skin brown spots area percentage was measured on Days 0, 28, and 56 using the VISIA‐CR system. (d) Representative images of fixed facial areas taken using the VISIA‐CR system, analyzed for brown spots using the Cross‐Polarized mode. Data are expressed as mean ± SEM. Statistical significance: ***p < 0.001 (within‐group changes in the PG group compared to the baseline value, paired *t*‐test); #*p* < 0.05, ##*p* < 0.01, ###*p* < 0.001 (between‐group changes between the PG and placebo groups at the same time, two‐way ANOVA).

Melanin levels in a fixed area on the subjects' cheeks were measured using the Mexameter MX18 probe on the CK‐MPA10 system. Additionally, full‐face skin brown spots area and skin brown spots area percentage were assessed using the Cross‐Polarized mode with RBX Technology on the VISIA‐CR system. Results showed significant reductions in melanin levels (Figure 5a), skin brown spots area percentage (Figure 5b), and skin brown spots area (Figure 5c) in the PG group compared to baseline values. Figure 5d shows representative images of skin brown spots captured by VISIA‐CR, highlighting a noticeable reduction in brown spots around the lips, forehead, and cheeks. Compared to the placebo group, after 56 days of product use, the PG group showed statistically significant improvements in these skin pigmentation metrics, indicating the effectiveness of the treatment.

### 3.5 Subjects' Subjective Self‐Assessment Survey

**FIGURE 6:**
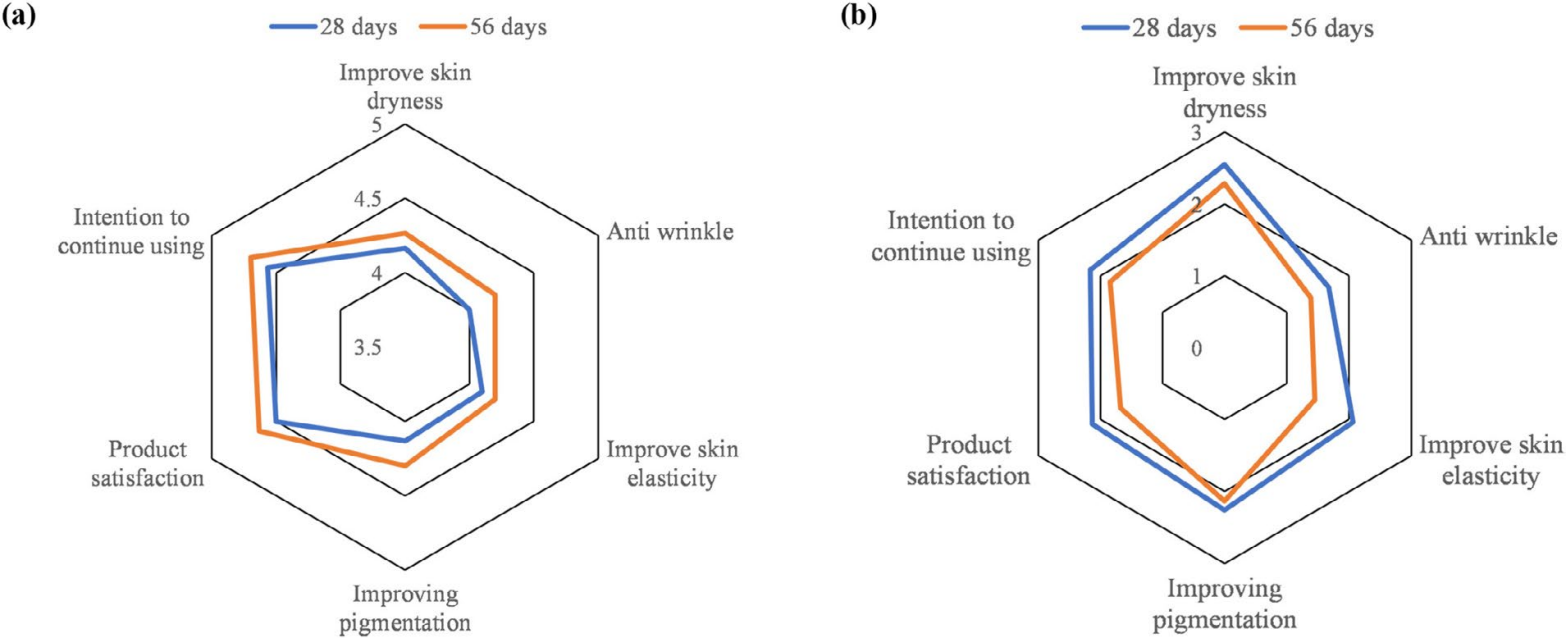
Subjective evaluation scores for skin improvements at 28 and 56 Days. (a) Average scores for various items reported by subjects in the PG group after 28 days (blue line) and 56 days (orange line) of product use; (b) Average scores for various items reported by subjects in the placebo group after 28 days (blue line) and 56 days (orange line) of product use.

Subjects' subjective experiences were evaluated and averaged after 28 and 56 days of product use (Figure 6). Results indicated that the PG group scored higher across all assessment items compared to the placebo group, with scores ranging from “somewhat satisfied” to “satisfied.” Moreover, the subjective scores of subjects after 56 days of use were higher than those after 28 days, indicating that PG had a noticeable and sustained improvement in the subjects' skin condition. In contrast, the placebo group had overall lower scores compared to the PG group, with ratings ranging from “very dissatisfied” to “dissatisfied.” Subjects in the PG group reported that the product was most effective in improving skin dryness, followed by brightening the skin, and provided satisfactory results in anti‐wrinkle effects and enhancing skin elasticity. These findings suggest that PG is likely to receive favorable consumer feedback in the market.

## 4 Conclusion

Our study focused on evaluating the antiaging effects of orally administered proteoglycans (PG) specifically on the facial skin of healthy middle‐aged individuals. In a 56‐day randomized, double‐blind, placebo‐controlled trial, we conducted comprehensive assessments of various skin parameters, including hydration, elasticity, roughness, wrinkle formation, melanin levels, and pigmentation, to explore the effects of PG on the skin of healthy subjects. No adverse reactions such as skin redness, itching, dryness, flaking, sensitivity, or gastrointestinal distress were observed in any subjects. Shigemitsu Kudo et al. investigated the toxicological profile of salmon nasal cartilage powder, including acute toxicity, genotoxicity, and repeated dose toxicity, using mouse models [13]. Additionally, Tatsuya Wada et al. recruited subjects who consumed 200 mg of Proteoglycan Complex 80 daily for 6 weeks with no clinically significant side effects [28]. The Japan Health & Nutrition Food Association recommends a daily intake of no more than 3 g [29]. These studies, alongside our findings, suggest that the daily intake of 20 mg in our study falls well within the safety margin and is unlikely to pose any significant health risks.

In this study, we demonstrated that PG intake significantly improves skin elasticity, hydration, roughness, and wrinkles—key parameters that are well‐established indicators of skin aging. Skin hydration reflects the degree of dryness and the water‐retention capacity of keratinocytes in the epidermal layer [30]. Skin elasticity, roughness, and wrinkles are indicators of the health of dermal collagen and elastin, as well as the vitality of dermal fibroblasts [31]. YO Tsuchiya et al. studied the absorption of PG in an isolated small intestine model, showing that PG can be absorbed through clathrin‐mediated endocytosis, with the highest transport efficiency observed in the jejunum [32]. Ikuko Kakizaki et al. analyzed the amino acid sequence of proteoglycans derived from salmon nasal cartilage and found that the G3 domain has a similarity to the human EGF‐like module [20], which is a key target for binding to human extracellular matrix proteins and stimulates EGF‐like proliferative activity in human dermal fibroblasts. Masahiro Sano and colleagues further supported this by showing that PG promotes the proliferation of dermal fibroblasts through the Erk1/2 phosphorylation pathway, like the effects of growth factors [33]. Although direct evidence on PG's absorption and transport to the dermis is limited, understanding these pathways is crucial. Elucidating these mechanisms poses a significant challenge for future research. On the other hand, a study by Asano et al. indicated that oral PG improves beneficial gut microbiota, including lactobacilli and short‐chain fatty acid‐producing bacteria [34]. Yusuke Okamoto's research further suggests that PG, with intact chondroitin sulfate and type II collagen structures, can specifically bind to L‐selectin, positively influencing immune regulation [35].

Our study also found that oral PG can improve symptoms related to photoaging, as evidenced by reductions in skin melanin content and brown spots. Hae Ran Lee et al. found that feeding mice with PG can improve UVB‐induced collagen degradation through the MAPK‐JNK signaling pathway, reducing inflammation and skin structure damage caused by photoaging [36]. Masashi Goto et al. discovered that PG can inhibit the inflammatory cytokine storm during the photoaging process, effectively reducing UVB‐induced erythema [37]. This UVB‐induced erythema, linked to delayed subcutaneous melanin production and pigmentation [38], highlights a potential mechanism for the observed reduction in pigmentation in our study subjects.

In summary, our research results indicate that a daily intake of 20 mg of PG is effective in improving skin elasticity, hydration, wrinkles, and reducing pigmentation in individuals aged 30–60. The subjects in our study also reported high satisfaction with the product, suggesting that PG could be a valuable addition to existing skincare and beauty food systems, offering a safe and effective antiaging skincare solution in a relatively short period. However, the limitations of this study, including the limited diversity of the subject sample and the incomplete exploration of mechanisms, point to areas for further research. Future studies should aim to confirm these findings in more diverse populations—considering factors such as age, ethnicity, and skin type—and to verify potential biological mechanisms to fully elucidate the benefits of PG for consumers.

## Author Contributions

**Xue‐dong Bai:** has made substantial contributions to the conception and design, acquisition of data, or analysis and interpretation of data; has been involved in drafting the manuscript or critically revising it for important intellectual content; and has given final approval of the version to be published. **Yu‐chen Liu** and **Sai‐ya Ge:** participated in the design of the methodology and made significant contributions to data curation and formal analysis. **Wei‐cheng Fei:** responsible for funding acquisition and resource allocation, project administration, and has contributed to the critical review, commentary, and revision of the manuscript. All authorshave participated sufficiently in the work to take public responsibility for appropriate portions of the content. All authors have reviewed and approved the final manuscript and agree to be accountable for all aspects of the work, ensuring that any questions regarding the accuracy or integrity of any part of the study are properly investigated and resolved.

## Ethics Statement

All authors confirm that the ethical policies of the journal, as noted on the journal's author guidelines page, have been adhered to and the appropriate ethical review committee approval has been received. All study procedures were conducted in accordance with the World Medical Association's (WMA) Declaration of Helsinki and its subsequent amendments (Ethical Principles for Medical Research Involving Human Subjects, adopted by the 18th WMA General Assembly, Helsinki, Finland, June 1964, with subsequent amendments).

## Conflicts of Interest

The authors declare no conflicts of interest.

## Data Availability

The data that support the findings of this study are available on request from the corresponding author. The data are not publicly available due to privacy or ethical restrictions.
